# Justice for Placebo: Placebo Effect in Clinical Trials and Everyday Practice

**DOI:** 10.3390/medicines12010005

**Published:** 2025-02-24

**Authors:** Nebojsa Nick Knezevic, Aleksandar Sic, Samantha Worobey, Emilija Knezevic

**Affiliations:** 1 Department of Anesthesiology, Advocate Illinois Masonic Medical Center, Chicago, IL 60657, USA; aca.smed01@gmail.com (A.S.); samantha.worobey@my.rfums.org (S.W.); emmaknezevic02@gmail.com (E.K.); 2 Department of Anesthesiology, University of Illinois, Chicago, IL 60612, USA; 3 Department of Surgery, University of Illinois, Chicago, IL 60612, USA; 4 Faculty of Medicine, University of Belgrade, 11000 Belgrade, Serbia; 5 Chicago Medical School, Rosalind Franklin University of Medicine and Science, North Chicago, IL 60064, USA

**Keywords:** placebo, nocebo, clinical trials, history, pain, clinical use

## Abstract

The placebo effect has been widely documented across various medical conditions, demonstrating its ability to influence both subjective and objective outcomes. Placebo responses can significantly improve symptoms in these different conditions, such as pain, Parkinson’s disease, depression, anxiety, and addiction. Psychological mechanisms, particularly the power of patient expectations, appear to play a central role, with neurobiological evidence supporting the activation of dopamine, endogenous opioids, and endocannabinoids in response to placebo interventions. Studies have demonstrated that placebo injections and more complex procedures, including sham surgeries, can produce therapeutic effects comparable to real treatments, particularly in pain management and neurological disorders. Moreover, placebo responses could be amplified when patients are aware of receiving treatment, as shown by research on open-label placebos and open versus hidden medical treatments. The effectiveness of 0.9% sodium chloride solution as a placebo in clinical trials is debated, with some studies indicating its potential to induce clinical improvements, though it may not be an ideal control in inflammatory pain conditions. Advances in neuroimaging have revealed that placebo treatments trigger tangible biological processes in the brain and body and are supported by psychological and physiological mechanisms that interact, suggesting real biological processes are involved in the observed effects. Overall, the growing understanding of placebo mechanisms suggests that incorporating placebo-based strategies, with patient awareness and appropriate ethical considerations, may offer significant potential for improving patient outcomes, particularly in chronic pain, mental health, and neurological conditions.

## 1. Introduction

**Figure 1:**
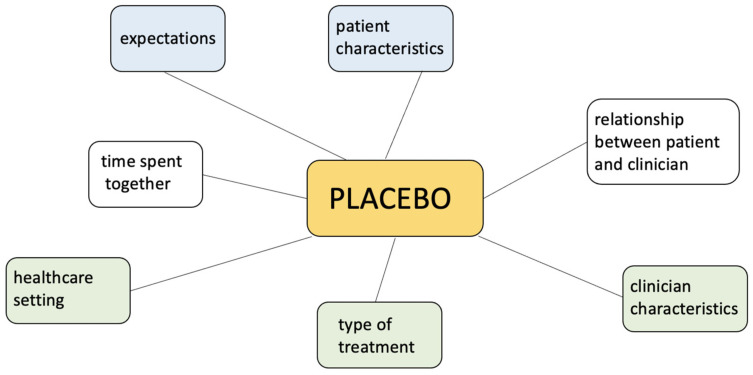
Factors influencing the placebo response.

The placebo has long been a source of intrigue and controversy in medicine. The placebo, traditionally defined as a pharmacologically inactive substance or “sugar pill”, has long been a cornerstone of clinical research, primarily serving as a control to measure the efficacy of active medical interventions. However, this simplistic definition belies the intricate psychological and physiological phenomena associated with placebo, which stem from the interplay between patient expectations, clinician–patient dynamics, and the healthcare environment (Figure 1). These factors can elicit tangible physiological responses, such as the release of different endogenous substances, leading to measurable symptom improvements. Neuroimaging studies reveal that placebo treatments activate brain regions involved in pain modulation, such as the prefrontal cortex and anterior cingulate cortex, while engaging endogenous pathways that underpin therapeutic outcomes [1].

This manuscript begins by highlighting the evolving role of placebos, historically regarded as inert substances, in modern medicine. The primary objective is to advocate for a broader understanding of placebo effects, emphasizing their neurobiological underpinnings and clinical evidence. This review aims to establish that placebos should no longer be viewed merely as controls or fake treatments in clinical trials but recognized as valid therapeutic modalities that leverage human interactions and the expectation of benefit to produce real, measurable changes in health. This review explores these multifaceted effects, emphasizing the placebo effect’s broader implications beyond clinical trials and advocating for its reframing as an activator of innate healing mechanisms rather than merely an inert comparator.

Figure 1 illustrates the key factors that contribute to the placebo response in medical and clinical contexts. These factors include patient characteristics, expectations, time spent with the clinician, the healthcare setting, the type of treatment provided, clinician characteristics, and the relationship between the patient and clinician. Each factor interacts dynamically to modulate the perceived efficacy of the treatment, even in the absence of an active pharmacological agent.

## 2. Methodology

This narrative review was conducted to explore the placebo effect and its mechanisms by synthesizing evidence from published studies. A literature search was performed in PubMed, Google Scholar and ScienceDirect databases, using key search terms “Placebo in clinical trials” “Placebo effect in pain”, “Placebo effect in Parkinson’s disease” “Placebo effect in depression”, “Placebo response”, and “Neurobiological mechanisms of placebo”. Boolean operators (AND, OR) were used to refine the search and ensure comprehensive results.

The initial search yielded 397 articles. After screening titles and abstracts, 104 studies were selected for full-text review. After eliminating duplicates and ensuring the relevance of the studies, we ultimately used 74 studies in our final synthesis. This selection was based on several key criteria: First, priority was given to studies directly addressing the placebo effect in specific conditions such as pain, Parkinson’s disease, and depression, ensuring that the review focused on the most relevant research for understanding the placebo effect in different clinical contexts. Second, more recent studies were prioritized, with preference given to publications from the last 5–10 years, though older studies were included if they provided foundational insights or were particularly influential. Additionally, randomized controlled trials (RCTs) were given particular priority, as they provide the highest level of evidence in clinical research, especially regarding placebo effects in clinical trials. Only peer-reviewed studies were included to ensure the quality of the sources, with special attention paid to those offering insights into the neurobiological mechanisms of the placebo response or those with clinical implications. Furthermore, the most relevant literature reviews were incorporated to ensure a comprehensive understanding of the placebo effect from various perspectives.

No formal quality assessment of included studies was conducted, given the narrative nature of this review. While systematic methodologies such as PRISMA were not employed, efforts were made to provide a broad yet focused synthesis of the topic.

## 3. History of Placebo

Historically regarded as an inert treatment, placebos were used in the early 19th century to placate patients, often administered in the form of bread pills or sugar water to appease symptoms without active medical ingredients [2]. However, the concept of the placebo shifted slightly in the 20th century, when the role of psychological and neurobiological mechanisms underlying placebo responses began to be more fully recognized [2,3,4,5].

The evolution of placebo use began in the 1930s when placebos began to be used as control groups in clinical trials, namely “Cold Vaccines: An Evaluation Based on A Controlled Study”, by Drs Diehl, Baker, and Cowan [6]. The recognition of the placebo effect as a clinical phenomenon gained prominence in the mid-20th century. During World War II, Dr. Henry Beecher observed the remarkable analgesic effects of 0.9% sodium chloride solution injections given to injured soldiers during the shortage of morphine [2,7]. Beecher’s insights and continued trials in sham procedures, later formalized in his groundbreaking 1955 paper, “The Powerful Placebo”, helped establish the placebo effect as a measurable and clinically relevant phenomenon [2,4,8,9]. He analyzed 15 RCTs and estimated that up to 35% of therapeutic effects in clinical practice could be attributed to placebo responses [10]. Subsequent studies have further demonstrated the placebo effect’s robust impact. In a 1959 RCT on internal mammary artery ligation for angina, patients undergoing the sham procedure reported similar improvements in symptoms as those who received the actual surgery [11]. These findings were pivotal in establishing placebo controls as a gold standard in clinical trials while also highlighting their therapeutic potential.

However, this did not ensure that placebo interventions would resume their previous role as therapeutic entities. The placebo effect was appreciated as a powerful psychological phenomenon, but its function has largely remained to be a means of comparison, used to create control conditions—deemed a “non-treatment”. However, the idea of the placebo as a “fake” or “inactive” treatment is a misnomer. Placebo interventions are not tricks or deceptions, but real therapeutic tools. These interventions utilize the power of patient–practitioner interactions, the sensory experiences of medical treatment, and the belief in the efficacy of the treatment to bring about measurable improvements in symptoms [12]. Importantly, placebo interventions have been found to create not only subjective improvements in patients but objective, physiologic changes as well [12,13].

Not only is this misnomer resulting in the underutilization of placebo as safe and effective medical tools, it is undermining clinical research, minimizing the effects of treatments that are being compared not to a true control, but a potential treatment in and of itself.

## 4. Placebo Effect in Different Conditions

Finnis et al. (2011) highlighted a broad spectrum of conditions where placebo effects have demonstrated efficacy, including pain, Parkinson’s disease, depression, anxiety, addiction, and improvements in various bodily systems and overall performance [13]. They found that patients with depression can experience nearly the same level of relief from a placebo as those treated with active medication. These findings suggest that conditions involving subjective outcomes, such as self-reported symptoms or perceptions of well-being, may be particularly susceptible to placebo responses. This is likely due to the psychological mechanisms underpinning the placebo effect, which can influence individuals’ perceptions and interpretations of their experiences, especially when objective measurement of outcomes is challenging. In Parkinson’s disease, placebo treatments are associated not only with subjective improvements but also significant objective improvements in motor control, often linked to the release of dopamine [12].

Seymour and Matthes found that placebo response in depression may be particularly pronounced due to the psychological mechanisms that mediate subjective experiences of well-being. Additionally, neurobiological changes such as neuroplasticity may underpin the placebo effect in depression, enhancing the therapeutic outcome through changes in brain function. These neuroplastic changes contribute to improving mood and reducing depressive symptoms by strengthening neural pathways associated with positive emotional regulation and stress resilience [14].

A meta-analysis by Vase and Wartolowska demonstrated that in many pain studies, including low back pain and chronic migraine, the placebo response is not only present across participants but also large enough to have clinical significance [15]. Such findings challenge conventional views of placebos as mere control interventions, showing instead that they hold therapeutic potential in their own right.

Hu et al. conducted a systematic review and meta-analysis of 60 randomized controlled trials (RCTs) to assess the impact of placebo effects in patients with musculoskeletal neck pain [16]. Their findings revealed that 38% of the pain reduction observed in patients treated with active interventions could be attributed to placebo or psychological effects. This highlights the significant role that placebo mechanisms play in pain management, even within the context of active treatment protocols [16].

Quattrone et al. reviewed 19 studies conducted between 2001 and 2015, demonstrating that placebo administration can elicit measurable neurobiological responses detectable through various imaging modalities, including PET scans, fMRI, deep brain stimulation, and rTMS. Notably, PET scan results showed dopamine release following placebo administration, emphasizing the physiological impact of psychological expectations [12]. Building on this, Lidstone et al. conducted a randomized, repeated-measures, two-day study involving 35 patients with Parkinson’s disease. Participants were informed that they had a 25%, 50%, 75%, or 100% chance of receiving real levodopa or a placebo. A PET scan performed one hour post-administration revealed dopamine release even with placebo administration. Interestingly, the highest dopamine release was observed in patients told they had a 75% chance of receiving the active drug, rather than those told they had a 100% likelihood [17]. This finding raises important questions about the impact of patient expectations on treatment efficacy and suggests that overpromising treatment outcomes may not always optimize therapeutic benefits.

Vollert et al. examined the effects of placebo injections in patients with rheumatoid arthritis. Their study found that placebo treatments led to a significant reduction in self-reported pain levels, as well as a decrease in inflammatory markers such as C-reactive protein (CRP) and erythrocyte sedimentation rate (ESR). These findings suggest that while placebo effects can influence subjective pain perception, they may also have an impact on underlying inflammatory processes in rheumatoid arthritis [18].

Below, Table 1 presents a summary of key findings from mentioned meta-analyses and reviews, emphasizing the diverse applications of placebo effects in clinical practice.

### 4.1. Beyond ”Sugar Pills”—Injections

While “sugar pills” have historically been the most common form of placebo intervention, recent studies suggest that more complex placebo procedures may be even more effective. Placebo injections, sham surgeries, and placebo implants have all been shown to produce significant clinical improvements, often comparable to real treatments.

**Figure 2:**
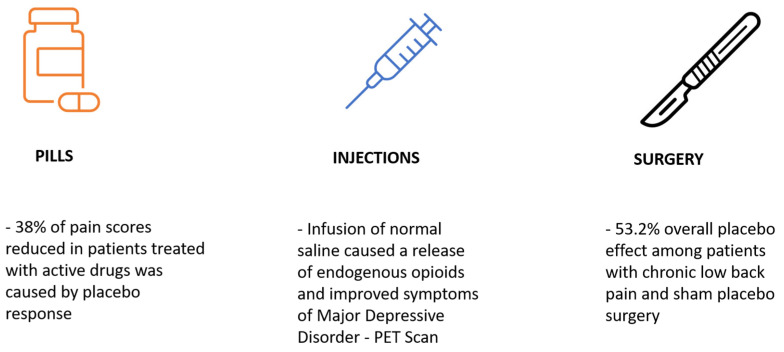
More complex medical treatments causing a higher placebo response.

More in-depth procedures mean more time with physicians, more sensory input, and a greater placebo effect (Figure 2). Alone, these components are effective in eliciting a placebo effect, research has shown that the effects are additive, thus more intensive placebo means more significant results [19].

There is another aspect to consider that may amplify the results of a more intensive placebo intervention: to receive an injection or undergo a procedure, a patient is putting themselves through a certain amount of pain, investing more of themselves in their outcome than simply swallowing a pill [20]. Additionally, it has been noted that patients believe that if they undergo a procedure, this direct physical alteration of their body will result in a significant change in functioning. Whether or not this is true, the belief itself produces relief [21]. This hierarchy is supported by the study by Kaptchuk et al., exploring sham acupuncture, finding that those who received sham acupuncture reported a greater reduction in arm pain compared to those receiving a placebo pill [22].

Tambiah et al. investigated the use of placebo (0.9% sodium chloride solution) and sham (dry needle) injections in knee osteoarthritis. Both placebo and sham groups showed significant improvements in pain and function, with improvements reaching a 10% minimum clinically important difference in pain and function scores [23].

Similarly, Vollert et al. assessed placebo injections in rheumatoid arthritis and found significant reductions in pain and inflammation. Patients receiving placebo injections had a 14-mm reduction in VAS pain scores at 12 weeks and a 1.16 mg/dL reduction in CRP levels, demonstrating that placebo injections can lead to both subjective pain relief and objective inflammation reduction [18].

Pecina et al. conducted a single-blinded RCT including patients with Major Depressive Disorder (MDD) who are not currently taking any medications. They were randomized into two groups. One group received an active placebo, and the other group received an inactive placebo for one week. Then after 3 days of washing out, the groups were switched for another week. Following that, they started open-label selective serotonin reuptake inhibitor (SSRI) therapy. The results showed that a one-week active placebo resulted in a significant decrease in depressive symptoms and the PET scan showed that an IV infusion of 0.9% sodium chloride solution caused the release of endogenous opioids (Figure 2). The study highlights the activation of the opioid system, showing that even a placebo intervention can trigger significant physiological changes, such as the release of endogenous opioids, which may contribute to the observed improvement in depressive symptoms [24].

### 4.2. Beyond ”Sugar Pills”—Surgeries

Unfortunately, the literature is severely limited in terms of comparing the efficacy of placebo medication with the efficacy of placebo surgery or device. In general, there is minimal use of placebo surgeries in clinical research due to ethical concerns. The findings that have been reported, and the theoretical framework of the placebo effect, suggest that the placebo effect could be amplified by going beyond pills [25]. We believe that research into this could produce remarkable results, and significant advancement in many treatment areas, especially pain, mental health, and other neurological areas. Given the significant burden these areas place on individuals and the healthcare system as leading causes of disability, suicide, and medical expenses in the United States [26,27,28], exploring innovative approaches to address this persistent challenge is imperative.

A systematic review of orthopedic procedures (including vertebroplasty, intradiscal electrothermal knee arthroscopy, and epicondylitis debridement) by Louw et al. analyzed 6 RCTs which included sham surgeries. The findings revealed that outcomes of sham surgeries were as effective as genuine surgery in improving pain and disability [21].

Wartolowska et al. conducted a systematic review examining the use of placebo controls in evaluating minimally invasive surgical procedures. The final analysis included 53 randomized controlled trials (RCTs), with 39 of these studies published after 2000, providing more recent data. The reviewed studies addressed various conditions, including migraine, obesity, GERD, sleep apnea, and others. The findings revealed that 72% of the studies reported improvements in both placebo and surgical groups, while 51% showed no statistical difference between sham and real surgery [29].

The authors questioned the ethics behind sham procedures, but given the efficacy of these trials and successful open-label placebo trials, what is more unethical—offering a sham surgery that is proven to be effective or continuing to push more dangerous yet not significantly more effective “real surgeries”.

Louw et al. reviewed six randomized controlled trials conducted between 2001 and 2012, involving 277 patients who underwent sham surgeries for conditions such as vertebroplasty, intradiscal electrochemical therapy, knee arthroscopy, and epicondylitis debridement [21]. This analysis demonstrated that sham surgery was as effective as true surgery in improving pain and disability in most cases, with only one study showing a significant difference between the groups. These findings highlight the potential for placebo effects to play a substantial role in surgical outcomes, raising important questions about the necessity of certain surgical interventions [21].

Jamjoom et al. conducted a systematic review investigating the placebo effect associated with sham spine procedures in patients with chronic low back pain [30]. The review included 17 studies with a total of 535 patients and 55 pain-scoring episodes. Their analysis revealed a significant reduction in pain scores in 21 of the 55 episodes, with an overall placebo effect of 53.2% (Figure 2). Notably, the placebo effect was observed in nearly half of the patients during the first six months following the sham procedure [30]. This finding is particularly compelling when compared to placebo effects observed with oral medications (38% improvement), injections, and even smaller surgical interventions, where placebo responses tend to be somewhat higher. These results suggest that even with larger surgeries, the psychological and placebo effects can be substantial, emphasizing the need to reconsider the traditional narrative surrounding the placebo effect. Rather than viewing it as a mere non-pharmacologically active substance or a “sugar pill”, it may be more accurate to view placebo responses as a means to activate the body’s innate healing potential. The degree to which each individual can access this potential may vary, but it could play a critical role in expediting recovery and addressing the challenges faced by patients.

Figure 2 illustrates the relationship between the complexity of medical interventions and the magnitude of placebo effects observed. These findings emphasize the enhanced placebo effects associated with increasingly complex treatment modalities.

### 4.3. Open vs. Hidden Medical Treatments

Evidence for this includes two experimental studies which demonstrated that hidden treatments such as injections and infusions for things such as pain, Parkinson’s management, and anxiety were significantly less effective than when patients were explicitly made aware treatment was being initiated [28,29].

A study involving 42 post-thoracotomy patients evaluated the effects of open versus hidden administration of morphine on pain management. Both groups received identical morphine infusions simultaneously; however, one group was informed they were receiving morphine, while the other group was not. Pain scores recorded one hour later revealed a statistically significant difference: patients aware of the morphine administration reported lower pain levels compared to those unaware [31]. In a follow-up segment of the study, the effects of open versus hidden interruptions of morphine infusion were examined. One group was informed their morphine infusion would be discontinued, while the other group was not notified. Pain scores rose more steeply in the group informed of the interruption. Additionally, by the 10th hour, 14 patients in the informed group requested additional morphine, compared to only six patients in the hidden interruption group [31].

The same study included 30 postoperative patients with elevated state anxiety, who were assessed during open versus hidden benzodiazepine administration. In the open administration group, patients were told they were receiving a benzodiazepine to reduce anxiety, whereas the hidden group was not informed. After two hours, the informed group demonstrated significant reductions in anxiety scores, while the hidden group exhibited no change in anxiety levels [32]. A further subsection of the study examined open versus hidden diazepam interruptions in 32 patients over 48 h. Initially, both groups received diazepam, but later, one group was informed their infusion would be stopped, while the other group experienced the discontinuation without being notified. Anxiety levels in the informed group increased significantly following the interruption, whereas the hidden group’s anxiety levels remained stable. The State-Trait Anxiety Inventory was employed to quantitatively assess anxiety states across both instances [31]. These findings highlight the critical role of patient awareness and psychological factors in modulating both pain and anxiety responses to pharmacological interventions.

A randomized controlled trial (RCT) was conducted with 10 patients with Parkinson’s disease, all of whom had implanted deep brain stimulation (DBS) electrodes. The study employed a between-subject design, where each participant underwent two days of stimulation manipulation, with each condition applied on separate days. The stimulation intensity was either overtly or covertly adjusted (decreased or increased), and the patients underwent movement tests under both conditions. The results indicated that open interruptions of DBS stimulation led to a significant reduction in movement velocity, while open increases in stimulation intensity resulted in a more pronounced improvement in movement velocity. These findings underscore the influence of both stimulation manipulation and patient awareness on motor performance in Parkinson’s disease [31].

Similar results were obtained even in healthy volunteers who received beta blockers or atropine. Twenty-four healthy volunteers were given propranolol and were split into two groups. One group knew what they were receiving, and the other group did not know. The open administration of propranolol produced a greater drop-in heart rate at 15 min when compared to the hidden administration [30]. In the second part of the study, 26 healthy volunteers were given Atropine which is known to increase the heart rate and were split into two groups. While both groups’ heart rates increased, the open administration of atropine produced a greater increase in the heart rate of the patients at 15 min when compared to those who were administered it hiddenly [31].

## 5. Differences in Placebo Effect in Clinical Trials vs. Everyday Practice

Recent studies have further explored the differences in placebo effects between clinical trials and everyday medical practice. A meta-analysis published in *Trials* examined placebo responses in randomized clinical trials, highlighting that the association between placebo-controlled effects and placebo responses diminishes when excluding trials with specific methodological issues [32]. Additionally, a systematic review in *Scientific Reports* assessed the impact of open-label placebos in clinical trials. The review found that open-label placebos—where patients are aware they are receiving a placebo—can elicit beneficial effects, suggesting that the therapeutic context and patient expectations play significant roles in treatment outcomes [33].

These findings underscore the complexity of placebo effects and suggest that while clinical trials aim to control for placebo responses, the therapeutic context and patient expectations in everyday practice can significantly influence treatment outcomes.

### 5.1. Open-Label Placebo

The ethical considerations surrounding placebo use are important in determining how placebos could be used even in clinical practice. Traditionally, placebos have been seen as deceptive treatments—patients were not told they were receiving an inert substance. However, studies have shown that open-label placebos—where patients are fully informed that they are receiving a placebo—can still produce significant improvements in symptoms. This suggests that the placebo effect does not require deception but can work with the patient’s understanding and belief in the treatment.

Belcher et al. performed a single-blinded RCT involving 131 patients to assess the effectiveness of conditioned open-label placebo alongside methadone in treating opioid use disorder over a 12-week period with five meeting time points [34]. Participants were divided into two groups: one received standard treatment, while the other received an open-label placebo along with their 90-day methadone regimen, preceded by brief education on the placebo effect. The study consisted of two phases: Phase 1, with once-daily placebo conditioning for two weeks, and Phase 2, with twice-daily placebo conditioning from weeks 3 to 12. Despite no significant differences in methadone dosage by day 90, the placebo group demonstrated significantly higher treatment retention rates and reported better sleep quality. These findings suggest that the use of conditioned placebo interventions may enhance patient engagement and overall well-being in opioid use disorder treatment [34].

### 5.2. Ethical Considerations and Informed Consent in Placebo Use

Ethics in medicine and research are based on informed consent, which is particularly important when it comes to the use of placebos, especially in open-label situations. The possibility to balance the therapeutic benefits of placebo responses with the ethical requirement of disclosure is unique in open-label placebo treatments, in which patients are made aware that they are receiving a placebo. Studies have shown that educating patients about the placebo nature of their medication does not always have a negative effect on its efficacy. In a randomized controlled trial, for instance, individuals with irritable bowel syndrome experienced a notable improvement in their symptoms even when they were aware that they were being given a placebo [35,36,37,38].

To address ethical concerns practically, clinicians should adopt transparent communication strategies that emphasize the potential benefits of placebo treatments. For example, explaining the psychological and physiological mechanisms of the placebo effect can foster trust and empower patients to participate actively in their treatment. Integrating shared decision-making frameworks ensures that patients fully understand and consent to the use of placebos while maintaining their autonomy [35,36].

Specific guidelines for the ethical integration of placebo treatments in clinical practice include:

- Patient Education: Inform patients about the placebo effect and its potential to improve symptoms, even without active medication [35,37].
- Transparency and Disclosure: Clearly state that the treatment involves a placebo, explaining its rationale as part of an evidence-based approach [37].
- Documentation of Consent: Obtain informed consent, emphasizing the voluntary nature of participation and outlining expected outcomes [38].
- Use Established Ethical Frameworks: The American Medical Association (AMA) encourages physicians to involve patients in decision-making by explaining that evaluating different types of medications, including placebos, can enhance understanding of the medical condition. This approach promotes shared decision-making and adheres to ethical standards [37].
- Continuous Monitoring: Continuously assess the patient’s response and adjust the treatment plan to ensure the placebo aligns with evolving clinical needs [35,36,37,38].

### 5.3. Evaluating the Appropriateness of 0.9% Sodium Chloride Solution as a Control in Clinical Trials

In clinical trials, including both FDA-regulated and investigator-initiated studies, 0.9% sodium chloride solution is commonly used as a control in injection-based interventions. Manchikanti et al. conducted a systematic review and meta-analysis to evaluate the efficacy of 0.9% sodium chloride solution as a placebo in epidural injection studies [39]. They analyzed data from 318 patients across eight randomized controlled trials comparing corticosteroids to 0.9% sodium chloride solution. The results showed no significant difference between the two treatments when analyzed directly. However, when a single-arm analysis was performed, both sodium chloride solution and corticosteroids demonstrated effects, with corticosteroids yielding slightly better outcomes in pain and functionality, as measured by the Oswestry Disability Index (ODI). Despite the sodium chloride solution showing some beneficial effects, this dual analysis did not reveal statistically significant differences, as both interventions improved outcomes [39]. This finding has contributed to the debate over the effectiveness of sodium chloride solution, with some insurance companies rejecting coverage for epidural injections with corticosteroids based on the notion that 0.9% sodium chloride solution alone can produce clinical improvements. Importantly, sodium chloride solution, when injected into areas with inflammation such as the epidural space, is not a true control due to its ability to wash out inflammatory mediators, providing a transient benefit. This suggests that 0.9% sodium chloride solution should not be used as a control in inflammatory pain conditions, and a true sham control should be employed instead. Similarly, Paget et al. conducted a multicenter, block-randomized, double-blind, placebo-controlled trial involving 100 patients with ankle osteoarthritis, comparing platelet-rich plasma (PRP) to sodium chloride solution placebo [40]. Despite the use of ultrasonography-guided intra-articular injections, no significant differences were observed over 26 weeks. This lack of effect may be attributed to the sodium chloride solution’s potential to wash out inflammatory mediators, which could have masked any beneficial effects of PRP [40].

### 5.4. Neurobiological Mechanisms

Over time, the perception of placebos has evolved from being considered inert treatments to active components in clinical research. This shift has been driven by advances in neuroscience, which have uncovered the biological processes underlying placebo effects. Understanding these mechanisms provides a scientific foundation for the historical observations of their therapeutic potential.

One of the most significant advances in placebo research has been the discovery of specific neurobiological pathways activated by placebo treatments. Recent studies using advanced brain imaging techniques such as positron emission tomography (PET) and functional magnetic resonance imaging (fMRI) have shown that placebo-induced changes in brain activity are not limited to psychological “tricks” but involve tangible biological processes. For example, a study published in *Nature Neuroscience* found that placebo treatments can modulate emotions [41], while research in *Brain* highlighted that placebo effects are genuine psychobiological phenomena attributable to the overall therapeutic context, with evidence showing that placebo effects can occur in clinical practice, even without the administration of a placebo [42].

**Figure 3:**
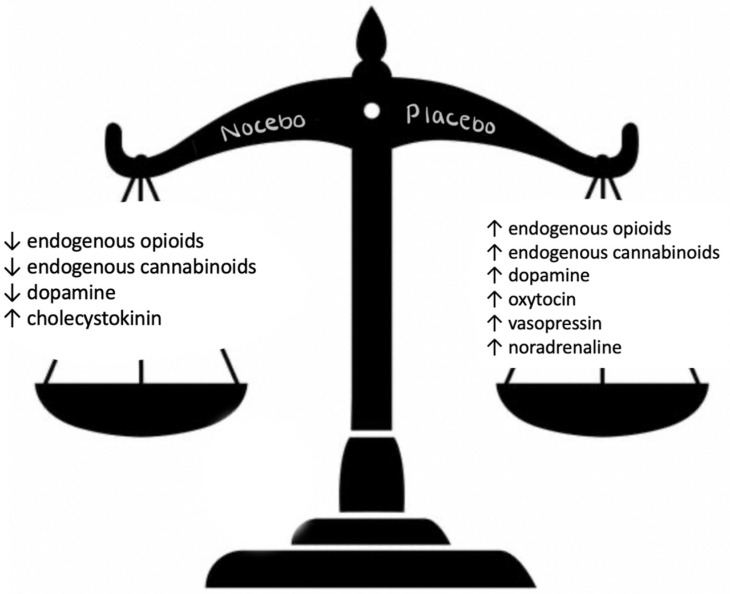
Neuromediators released during the placebo and nocebo response.

One notable study demonstrated that placebos activate the nigrostriatal pathway, a critical component of the brain’s dopamine system. Finnis et al. found that patients who believed they were receiving dopamine-enhancing medication experienced a real release of dopamine [13]. Nezer et al. examined how placebo treatments affect brain systems involved in affective processing, revealing that placebo treatments led to significant analgesia in response to conditioned thermal pain, which also generalized to unconditioned mechanical pain. This suggests that placebo-induced changes in brain activity extend beyond psychological factors, involving tangible biological processes [43]. Moreover, placebo treatments in patients with chronic pain have been shown to trigger the body’s natural pain-relieving systems, such as endogenous opioids and endocannabinoids. Other neuromediators, such as oxytocin, vasopressin, and noradrenaline, are also released during a placebo response (Figure 3) [20].

Furthermore, in patients with Parkinson’s disease, placebo treatments have been shown to activate the dopaminergic system, offering insights into the neurobiological mechanisms of placebo effects. In a study on Parkinson’s patients, placebo treatments induced dopaminergic responses, leading to symptom improvement and an increase in dopamine release, particularly in regions involved in motor control [44].

Placebo-induced pain modulation, as revealed by a meta-analysis by Atlas and Wager, consistently shows reductions in brain activity within regions crucial for pain processing, such as the left anterior, mid, and posterior insula, left putamen, and left amygdala. Simultaneously, placebo effects are associated with increased activity in areas involved in cognitive and affective regulation, including the pregenual anterior cingulate cortex (pgACC), medial orbitofrontal cortex (mOFC), ventromedial striatum, and anterior insula. These findings highlight the dual role of placebo in both reducing pain perception and enhancing regulatory mechanisms through expectancy-driven processes [45]. Additionally, a meta-analysis by Yeung et al. found that placebo treatment significantly improved perceived sleep onset latency, total sleep time, and global sleep quality in insomnia patients. However, no effect was observed on objective sleep onset latency, likely due to limited data. The placebo effect was consistent across various subjective measures, emphasizing the importance of subjective outcomes in insomnia treatment [46].

A study by Puviani and Rama proposed a quantitative model suggesting that the placebo response is driven by unconditioned stimulus (UCS) revaluation. This model indicates that the brain updates its response to a substance based on new information, contributing to the placebo effect [47].

These findings clearly demonstrate that placebos are not mere “shams” or “tricks”. The placebo effect transcends mental factors and involves physical processes in the brain and body.

Patient perception and expectations are fundamental to the efficacy of placebo treatments. Numerous studies have confirmed that positive expectations significantly enhance the placebo response, particularly in conditions involving pain management, depression, neurological function, and surgical recovery [17,42,48]. In a systematic review of surgical interventions, Vase and Wartolowski found that when patients expect their surgery will be successful, they are more likely to report pain reduction, both in sham and surgery groups [15].

A 2014 study by Kam-Hansen et al. demonstrated that the labeling of a treatment significantly influences its effectiveness, with both placebos and real medications showing improved efficacy when labeled “positively” (labeled with the name of a known medication, Maxalt). Further supporting the power of expectation, they found that Maxalt labeled as a placebo was no more effective than a placebo labeled as Maxalt [49].

Frisaldi et al. recently performed a pilot correlational study consisting of patients beginning immunosuppressive therapy for myasthenia gravis. Seventeen patients were assessed at baseline to determine their quantitative myasthenia gravis score (QMG), as well as their expected side effects and state anxiety. The study found that state anxiety moderated pre-treatment negative expectations, which were correlated with the fulfillment of those side effects [47].

Placebo treatments do not rely solely on conscious expectations but also on unconscious associations and expectations. There is an inherent belief that when seeking medical services, one’s health will improve. This expectation is shaped by sensory cues and behaviors commonly associated with medicine—signals to the brain indicating that treatment is imminent. Clinical settings, with their characteristic sights, sounds, and smells, interactions with professionals, and medical attention, have the power to activate the body’s natural healing processes [19,20,50].

The placebo effect does not operate in isolation; it can enhance the effectiveness of active treatments. The placebo effect arises from patient–practitioner interactions, sensory experiences, and the patient’s belief in the treatment process. By understanding and leveraging these factors, we can enhance the effects of both placebo and true medications, which could lead to reductions in medication doses, shorter treatment durations, increased patient safety, fewer side effects, and even reduced costs [33,45,49]. The effect of the placebo comes from patient–practitioner interactions, sensory experiences, and the patient’s belief in the treatment process. By understanding, and utilizing these factors, we can heighten the effects of both placebo and true medication, it is truly a shame we have not already carried this out. It could allow for reductions in medication doses and treatment duration, among other potential changes, leading to increased patient safety, decreased side effects, and even decreased costs [51,52].

Figure 3 depicts the contrasting neuromediator profiles associated with the placebo and nocebo responses, represented as a balance scale. The placebo response is characterized by an increased release of endogenous opioids, endogenous cannabinoids, dopamine, oxytocin, vasopressin, and noradrenaline. In contrast, the nocebo response is associated with reduced levels of endogenous opioids, endogenous cannabinoids, and dopamine, accompanied by increased release of cholecystokinin. These neuromediators contribute to the physiological and psychological effects observed during placebo and nocebo responses.

## 6. Nocebo Effect

The nocebo occurs when patients are told or may believe negative effects will come from treatment and thus they go on to experience them, whether there is a scientific reason for it or not. This is illustrated in a review of 73 clinical trials of migraine analgesics by Benedetti et al. in which they found that patients who received a placebo reported high rates of side effects, and these side effects were specifically those which researchers told them may occur [53]. Additionally, some of these side effects were only reported by those receiving placebo, not treatment [48]. Similarly, Rief et al. performed a systematic review of SSRI and TCA clinical trials and found that types of side effects reported by placebo groups mirrored those reported by treatment groups and that the structure of physician assessment of side effects greatly impacted the rates of side effects reported in placebo groups [54].

Additionally, studies addressing the biological mechanisms behind nocebo hyperalgesia, have illustrated through neuroimaging and pharmacological studies that nocebos have the power to activate cholecystokinin and inhibit endogenous opioids, cannabinoids, and dopamine (Figure 3) [55,56].

Patients who are anxious about chemotherapy may experience nausea and vomiting even before the treatment begins, particularly when receiving infusions like 0.9% sodium chloride solution. The infusion process itself can trigger a physiological and psychological response that mimics or amplifies the effects of chemotherapy, even when no active chemotherapy agents are involved. This phenomenon has been documented in various studies, where patients, particularly those who are apprehensive or have a history of motion sickness, may experience nausea due to the stress and anticipation of the treatment rather than the chemotherapy itself. Interestingly, some patients report nausea and vomiting during 0.9% sodium chloride solution infusion, which is typically used as a control in chemotherapy studies, underscoring the powerful impact of psychological factors on these symptoms. This suggests that the mere act of receiving an infusion, especially in a context laden with anxiety or negative expectations, may be enough to trigger the body’s nausea response. As such, managing these anticipatory symptoms becomes an important aspect of pre-treatment care for chemotherapy patients [57].

To mitigate such effects, healthcare providers often administer antiemetic medications or other preventative measures even before chemotherapy starts, highlighting the complexity of managing chemotherapy-induced nausea and vomiting (CINV).

## 7. Placebo vs. Nocebo

In comparing placebo and nocebo effects, a key distinction lies in their clinical prevalence and impact. While both effects are influenced by patient expectations, the clinical outcomes of these phenomena are often quite different. Placebo effects tend to lead to perceived improvements in symptoms due to the belief in the treatment’s efficacy, even when the treatment is inert. This positive expectation can trigger beneficial physiological responses, often aligning with the patient’s initial perceptions of the treatment. On the other hand, nocebo effects are typically associated with negative outcomes, where patients experience adverse symptoms due to the expectation of harm, despite receiving no active therapeutic intervention [58,59].

The nocebo effect is often underappreciated, as patients may fail to recognize or report symptoms that arise from their negative expectations. However, its clinical consequences can be profound, especially in chronic conditions or in cases where patients experience heightened anxiety during treatment. These effects can significantly influence treatment adherence and may even lead to treatment discontinuation or worsened symptomatology [60,61,62].

While placebo effects can contribute to patient satisfaction and treatment adherence, nocebo effects can have the opposite effect, potentially leading to treatment discontinuation or exacerbation of symptoms [58,59].

These phenomena underline the importance of clear communication and patient education in clinical settings. By providing transparent information about treatment expectations and outcomes, clinicians can help manage both placebo and nocebo effects, potentially enhancing therapeutic efficacy while minimizing adverse experiences.

## 8. Challenges with Placebo

Placebo effects may lead to falsely deeming treatments as ineffective [13,51]. In clinical trials, especially those focused on pain management, placebo groups are often required to compare the effects of medications, particularly for FDA approval [63,64]. However, pharmaceutical companies frequently encounter challenges when placebo responses are unexpectedly high [64]. One crucial factor that should be considered is the difference in the level of interaction between clinical trial participants and healthcare providers compared to routine patient visits. In clinical trials, patients typically receive more time and attention, whether it is during the consent process, frequent clinic visits or additional diagnostic tests such as blood draws or EKGs. This heightened level of interaction may not only influence the therapeutic outcomes but also contribute significantly to placebo responses [65].

In contrast, regular healthcare visits often involve less time with the physician, due to the pressures of the healthcare system and the financial constraints, such as reduced reimbursements for pain procedures [66]. The extended time and focused attention that clinical trial participants receive could have a substantial impact on their health outcomes, and this variable should be factored into the evaluation of trial results. It underscores the importance of considering these contextual factors when interpreting placebo responses and assessing treatment efficacy in clinical trials [32,64,67].

## 9. Discussion

Placebos have traditionally been viewed as pharmacologically inert substances or “sugar pills”, used primarily as controls in clinical trials. However, a growing body of evidence demonstrates that placebo effects stem from complex psychological and neurobiological processes, engaging the body’s innate healing mechanisms. This review emphasizes the need to reconsider the role of placebos in both research and clinical practice, moving beyond their conventional understanding.

One of the most compelling aspects of placebo research lies in its potential to reduce the reliance on invasive treatments or pharmacological interventions, minimizing side effects while optimizing patient outcomes. This is particularly relevant in pain management and other conditions where subjective experiences significantly influence treatment efficacy. However, ethical concerns remain a barrier to widespread adoption, particularly regarding informed consent and the potential perception of deception. Addressing these concerns through transparency and patient education is critical to ensure ethical implementation.

The variability in placebo responses, influenced by factors such as patient characteristics, treatment context, and the clinician–patient relationship (as illustrated in Figure 1 and Figure 3), highlights the need for further investigation. These individual and contextual differences provide opportunities to tailor placebo interventions for maximum benefit, but they also introduce challenges in standardizing and scaling these approaches.

A key area for future exploration involves complex placebo interventions, such as sham surgeries and devices, which have shown promise in some studies but require rigorous ethical scrutiny and validation. Additionally, the role of placebos in clinical trial design warrants careful consideration. Thoughtful selection of control groups that account for placebo effects is essential to accurately assess the efficacy of new treatments while recognizing the therapeutic potential of placebo mechanisms themselves.

By expanding our understanding of placebo effects within clinical and research frameworks, there is potential to enhance their therapeutic benefits. Placebos are valuable tools in research, and when contemplated, may offer additional benefits in patient care, particularly in managing symptoms where other treatments have limitations. Embracing their dynamic, patient-specific nature opens new possibilities for their application, not only in research settings but also in routine medical practice. This paradigm shift challenges the traditional view of placebos and invites a broader discussion on how best to utilize them ethically and effectively in modern healthcare.

## 10. Integration of Placebo Findings into Clinical Practice

The potential of placebo effects to improve patient outcomes offers significant opportunities for integration into clinical practice. One actionable strategy is enhancing patient–provider communication. Strong therapeutic alliances characterized by empathy, active listening, and transparent communication have been shown to positively influence treatment outcomes through improved patient expectations and trust. For example, studies that highlight patient-centered communication foster greater adherence to treatment and amplify therapeutic benefits, including placebo responses [68].

Another approach involves managing patient expectations. Explicitly discussing the potential benefits of a treatment, even with uncertain outcomes, has been shown to enhance the placebo effect by fostering positive expectations. Research demonstrates that positive framing of treatment efficacy significantly improves clinical outcomes, even in cases where the treatment is pharmacologically inert [69]. Conditioning mechanisms can also offer a promising route for clinical application in practice. Pairing active medications with inert substances, such as placebo pills, can lead to patients associating the placebo with therapeutic effects over time. This approach has shown particularly effective results in reducing doses of medication in chronic conditions like Parkinson’s disease and hypertension without compromising the efficacy of the therapy [70].

Additionally, postoperative recovery represents another context in which placebo effects can be leveraged. For instance, placebo interventions have been shown to reduce postoperative pain and accelerate recovery when combined with standard care protocols [71]. Placebo responses are also critical in clinical research settings where managing and understanding placebo effects can enhance the study design and possibly the interpretation of the efficacy of the treatments [72].

### Limitations of Placebo Research

Placebo research faces several limitations and potential biases that can significantly affect the validity of results. One major challenge is the difficulty in controlling for all variables influencing outcomes, such as patient expectations, physician communication, and psychological factors. Patient expectations, in particular, can lead to a placebo effect, where positive beliefs about treatment contribute to perceived improvements in health. Conversely, negative expectations can lead to the nocebo effect, wherein adverse outcomes or symptom exacerbation occur due to negative beliefs or anticipations about treatment. Separating the nocebo effect from other psychological or physiological influences is particularly challenging [58]. Another issue is the risk of bias in placebo trials. Biases can arise from researcher expectations, patient selection, and study design, potentially skewing results and leading to misleading conclusions about placebo efficacy. Additionally, response bias in trials with patient-reported outcomes further complicates the interpretation of placebo effects. Factors such as differential co-interventions, patient dropouts, publication bias, and outcome reporting bias can also introduce distortions in placebo research. The inherent non-blinded comparison between placebo and no-treatment groups often serves as an approximate and fairly crude reflection of the true effects of placebo interventions [73]. Furthermore, ethical concerns surrounding placebo use in controlled trials, particularly when effective treatments are available, complicate interpretation. Withholding an active treatment for the sake of a placebo can undermine patient trust and the validity of the study, raising significant ethical questions about informed consent [74].

These limitations emphasize the need for careful study design, transparency, and ethical considerations to ensure the accuracy and integrity of placebo research.

## 11. Conclusions

Placebos are powerful tools in medicine, capable of harnessing the mind-body connection to improve patient outcomes, particularly in symptom-driven conditions like pain. By engaging psychological and neurobiological mechanisms, the placebo effect may contribute to reducing the need for invasive treatments or pharmacological interventions in certain contexts. However, ethical concerns regarding transparency and patient autonomy must be addressed through informed consent and education. The variability in placebo responses highlights the need for further research into their mechanisms and tailored applications. Future studies should address key gaps in placebo research, such as understanding the individual and contextual factors influencing placebo effects, refining ethical frameworks for their use, and exploring complex placebo interventions like sham procedures. Methodological advancements, including neuroimaging and large-scale longitudinal studies, are essential to deepen our understanding and optimize placebo use. By reframing placebos as dynamic and patient-specific phenomena, we can unlock their full therapeutic potential, advancing both clinical practice and research.

## Figures and Tables

**Table 1:** Key findings from studies on the placebo effect in clinical practice.

Study	Key Findings	Area of Focus
Finnis et al. [13]	Placebo effects are effective across a broad range of conditions, with patients with depression experiencing relief similar to active medication. Conditions with subjective outcomes, like depression, are particularly susceptible to placebo responses	Efficacy of placebo in various conditions, including depression
Seymour and Matthes [14]	The placebo response in depression is pronounced due to psychological mechanisms and neuroplasticity, enhancing therapeutic outcomes through brain function changes	Placebo effects in depression and neuroplasticity
Vase and Wartolowska [15]	The placebo effect is significant in various pain studies, including migraine and low back pain, with clinically meaningful impacts	Effectiveness of placebo in pain management
Hu et al. [16]	38% of pain reduction in patients with neck pain was attributed to placebo or psychological effects	Role of placebo in musculoskeletal pain
Quattrone et al. [12]	Placebo treatments induce measurable neurobiological responses, including dopamine release in Parkinson’s disease	Neurobiological mechanisms of placebo
Vollert et al. [18]	Placebo injections in rheumatoid arthritis reduce both pain and inflammatory markers	Role of placebo in inflammatory conditions
Benedetti et al. [7]	Placebos can modulate emotions through measurable changes in brain activity	Emotional modulation through placebo

## Data Availability

No new data were created or analyzed in this study. Data sharing is not applicable to this article.
